# Effectiveness of a Couple-Based HIV and Sexually Transmitted Infection Prevention Intervention for Men in Community Supervision Programs and Their Female Sexual Partners: A Randomized Clinical Trial

**DOI:** 10.1001/jamanetworkopen.2019.1139

**Published:** 2019-03-29

**Authors:** Nabila El-Bassel, Louisa Gilbert, Dawn Goddard-Eckrich, Mingway Chang, Elwin Wu, Sharun Goodwin, Richard Tibbetts, Maria Almonte-Weston, Timothy Hunt

**Affiliations:** 1 Social Intervention Group, Columbia University School of Social Work, New York, New York; 2 New York City Department of Probation, New York, New York; 3 Bronx Community Solutions, Center for Court Innovation, Bronx, New York

## Abstract

**Importance:**

In the United States, the prevalence rates of HIV and sexually transmitted infections (STIs) are higher among individuals in community supervision programs (CSPs) than in the general population. However, to date, no couple-based HIV or STI prevention interventions have been implemented for the large number of men in CSPs.

**Objective:**

To determine the effectiveness of a 5-session couple-based prevention intervention, compared with a 1-session counseling, testing, and referral (CTR) program, in reducing HIV and STIs as well as condomless intercourse among men in CSPs and their female sexual partners.

**Design, Setting, and Participants:**

A randomized clinical trial was conducted from July 11, 2013 (first recruitment), through May 17, 2016 (last randomization). Participants were drug-involved men mandated to a CSP and their female sexual partners (n = 230 couples or 460 individuals). Participants were recruited from various CSP sites in New York, New York, and randomized into either the PACT (Protect and Connect) intervention condition or the HIV CTR control condition (n = 115 couples or 230 individuals in each arm). Analysis of behavioral outcomes used an intent-to-treat approach. Statistical analyses were conducted from November 1, 2017, through June 1, 2018.

**Main Outcomes and Measures:**

Self-reported data on sexual behaviors in the past 90 days were used to assess behavioral outcomes at all time points. Biomarkers were collected at baseline and 12 months, and behavioral outcomes were collected at baseline and 3, 6, and 12 months.

**Results:**

A total of 230 couples (460 individuals) were included. The mean (SD) age of participants was 35.0 (12.8) years, and most participants (341 [74.1%]) self-identified as black or African American race/ethnicity. Of the 18 new cases of STIs identified at the 12-month assessment, 10 came from the PACT arm and 8 from the HIV CTR control arm. Compared with the control participants, PACT participants had 33% fewer acts of condomless vaginal and/or anal intercourse with their main partner (incidence rate ratio [IRR], 0.67; 95% CI, 0.45-0.99; *P* = .04), 70% fewer acts with other partners (IRR, 0.30; 95% CI, 0.12-0.74; *P* = .009), and 40% fewer acts with all sexual partners (IRR, 0.60; 95% CI, 0.42-0.85; *P* = .005) over the entire follow-up period. In addition, PACT participants were less likely to report being under the influence of drugs or alcohol the last time they had vaginal and/or anal intercourse with their study partners (odds ratio, 0.55; 95% CI, 0.31-0.96; *P* = .04) and had 26% fewer sexual partners in the past 90 days (IRR, 0.74; 95% CI, 0.61-0.88; *P* = .001). At 12 months, HIV and STI incidence did not differ significantly between the 2 arms.

**Conclusions and Relevance:**

The PACT intervention appeared to reduce risky sexual behaviors, such as condomless intercourse; this finding suggests that a couple-based HIV and STI prevention intervention program may curb the burgeoning HIV epidemic in CSPs.

**Clinical Trial Registration:**

ClinicalTrials.gov identifier: NCT01690494

## Key Points

**Question:**

Is a 5-session prevention intervention for HIV and sexually transmitted infections effective in reducing sexual risk behaviors among men in a community supervision program and their main female sexual partners?

**Findings:**

In this randomized clinical trial of 230 couples, participants randomized to the 5-session couple-based prevention intervention arm reported significantly fewer incidences of unprotected sex, fewer sexual partners, and fewer sexual activities with other partners compared with participants randomized to a 1-session counseling, testing, and referral program.

**Meaning:**

Couple-based HIV and sexually transmitted disease prevention interventions appeared to have a substantial effect on reducing risky sexual behaviors.

## Teaser

This randomized clinical trial compares the effectiveness of a 5-session couple-based intervention vs a 1-session counseling, testing, and referral program in reducing HIV, sexually transmitted infections (STIs), and sexual risk behaviors among men in community supervision programs and their female sexual partners.

## Introduction

During 2016, nearly 7 million adults were involved in the US criminal justice system,^1^ with an estimated 4 537 100 undergoing community supervision (through probation, parole, community court, or alternative-to-incarceration program), which accounts for most of the adult correctional population in the United States.^1^ Studies have found HIV prevalence rates as high as 13% to 17% among individuals in community supervision programs (CSPs), which are higher than the rates among those in correctional facilities.^2,3,4^ Studies of men in CSPs have found elevated rates of sexually transmitted infection (STI) and multiple sexual partners as well as low levels (ranging from 10% to 23%) of condom use with main or other partners.^2,5,6^

Systematic reviews indicate that couple-based interventions help HIV-negative partners to stay negative and encourage HIV-positive partners to stay healthy.^7,8,9^ Couple-based interventions have been shown to be efficacious in increasing condom use and decreasing the number of sexual partners^10,11,12^; improving condom use intentions, condom use self-efficacy, proper technical use of condoms,^13,14^ and couple communication and negotiation skills^13^; and reducing drug use, problem drinking, and overdose.^15^ In addition, couple-based HIV interventions have been found to increase HIV testing rates and initiation and adherence to antiretroviral treatment.^14^ However, the existing number of couple-based interventions is low: the Centers for Disease Control and Prevention’s HIV/AIDS Prevention Research Synthesis Project for evidence-based interventions identified only 2 of 84 evidence-based interventions that addressed couples.^9^ Furthermore, to date, no couple-based HIV or STI interventions have been implemented for the large number of men in CSPs, to our knowledge.^7^

To address this critical gap in couple-based HIV intervention research, we conducted an effectiveness randomized clinical trial (RCT) of the PACT (Protect and Connect) HIV and STI intervention focusing on men recruited from CSPs and their main female sexual partners. The PACT intervention, adapted from the evidence-based couple-based interventions we developed,^4,10,11,12,16^ is tailored for men in CSPs and addresses the unique stressors that they encounter in protecting themselves from HIV or STIs. We hypothesized that couples in the 5-session PACT HIV and STI arm, compared with those in the 1-session PACT HIV counseling, testing, and referral (CTR) control arm, would report fewer acts of condomless vaginal and/or anal intercourse (primary behavioral outcome) and would be less likely to be HIV positive and/or have positive test results for STIs (ie, chlamydia, gonorrhea, or trichomoniasis) over the 12-month follow-up period.

## Methods

### Setting

This RCT was conducted from July 11, 2013 (first recruitment), through May 17, 2016 (last randomization), among men recruited from various CSP sites in New York, New York, and their main female sexual partners. The institutional review boards of Columbia University and the Center for Court Innovation approved all trial protocols (Supplement 1). All participants provided written informed consent. This study followed the Consolidated Standards of Reporting Trials (CONSORT) reporting guideline.

### Study Design

We used a 2-arm effectiveness RCT. The PACT intervention condition consisted of 5 HIV and STI risk-reduction sessions. The first session was 45 minutes long, and the next 4 sessions were 90 minutes each. The HIV CTR control condition consisted of one 45-minute session. Power analyses showed that a sample size of 96 couples per study arm was necessary to achieve 80% power to detect a clinically meaningful 50% reduction in cumulative STI incidence and 90% power to detect significant improvement in behavioral outcomes. Taking into account a 15% to 20% attrition during the follow-up period, we set the target sample size at 113 to 120 couples per study arm. The CONSORT flowchart is presented in the Figure.

**Figure.**
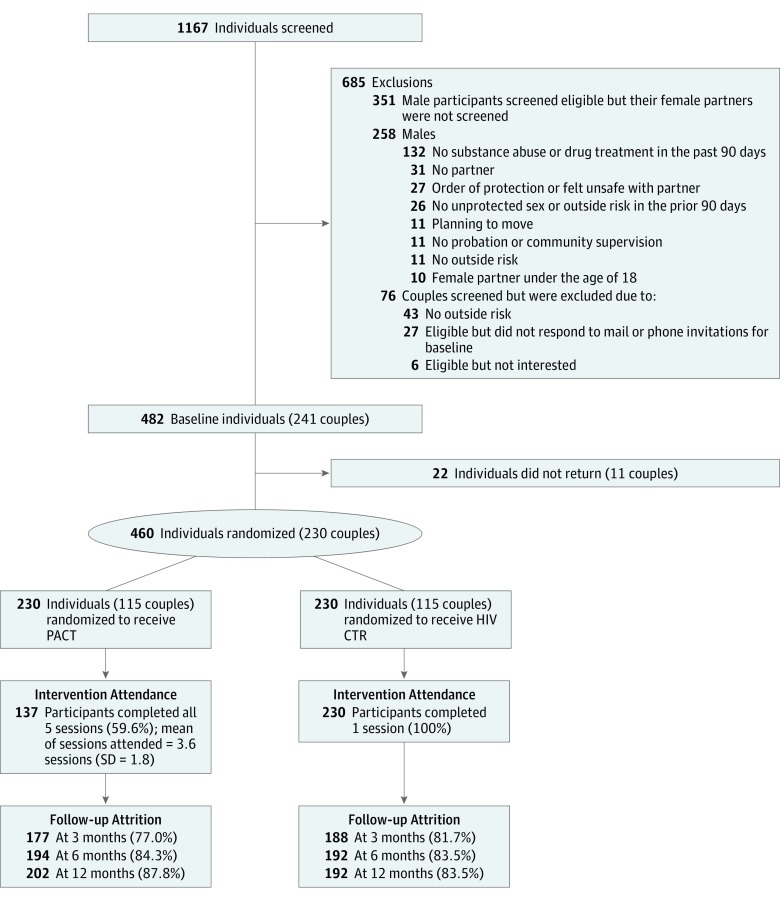
CONSORT Flow Diagram CTR indicates counseling, testing, and referral; PACT, Protect and Connect.

### Recruitment and Eligibility

Research assistants routinely handed out study announcement flyers to male clients at the CSP sites. If clients expressed interest, the research assistants completed informed consent procedures and conducted a screening interview to determine study eligibility (eAppendix in Supplement 2). If the potential male participant met the eligibility criteria, he was asked to invite someone whom he considered his main female sexual partner to participate. The female partner then underwent the consent procedure and was screened for eligibility. If the couple met the criteria, they were scheduled for a baseline assessment, which typically took 90 minutes to complete.

### Randomization and Masking

The computer-generated randomization algorithm was designed to randomly assign couples to 1 of 2 arms and to balance the number of couples per study arm through an adaptive, biased-coin procedure.^17^ We were all masked to treatment assignment until the final 12-month follow-up assessment was completed on July 7, 2017. Data were locked on September 30, 2017, and the study arms were then unmasked. One of us (E.W.), the designer of the randomization program, was involved in the statistical analysis but not in conducting the trial.

### PACT Intervention and HIV CTR Control

Intervention sessions for both arms were delivered in real-world CSP settings. The PACT intervention is guided by social cognitive theory,^18^ an ecological framework,^19^ and motivational interviewing skills.^20^ The Box contains the core elements of the intervention: (1) couple-based HIV CTR, (2) disclosure of drug and sexual risks; (3) couple communication, negotiation, and problem-solving skills; (4) technical condom use skills; (5) strategies for identifying and reducing personal risks, such as unsafe injections; (6) biomedical HIV-prevention strategies (HIV treatment as prevention, postexposure prophylaxis, and pre-exposure prophylaxis); (7) linkage to HIV, STI, and substance use treatment; (8) reproductive health issues; (9) risks and experiences of sexual coercion; (10) opioid overdose response and prevention; (11) informal social support; and (12) couple goal setting to increase protective behaviors.

Box. **Core Elements of PACT Intervention**

Five Risk to Reduction Sessions With Rapid Oral HIV CTR for PACT Intervention Condition

1. Provision of individualized health risk assessment, rapid oral HIV testing, and pretest and posttest counseling and referral to care
2. Disclosure of drug and sexual risks
3. Enhancement of the couple’s communication skills to enhance understanding of and commitment to healthier choices
4. Enhancement of condom technical skills, and addressing barriers to use of and access to female and male condoms
5. Identification and reduction of personal risks associated with drug use, including HIV risk of sharing syringes and decision to making about protected sex and medication adherence
6. Education on options for biomedical HIV prevention strategies, including HIV treatment as prevention, PEP, and PrEP
7. Linkage to HIV, STI, and substance use treatment
8. Discussion of reproductive health issues
9. Discussion of risks and experiences of sexual coercion
10. Education on overdose response and prevention, including knowledge and skills to use naloxone hydrochloride
11. Enhancement of social support for risk reduction, including identifying and setting goals to strengthen support from family and friends
12. Goal setting to increase protective behaviors (HIV testing, ART, and PrEP adherence) and decrease risky behaviors for HIV or STIs and overdose

One Session With Rapid Oral HIV CTR Plus Service Referral for Control Condition

1. Provision of individualized health risk assessment, rapid oral HIV resting, and pretest and posttest counseling and referral to care
2. Identification of current use of case management services identified and need for additional social and health services
3. Provision of a resource manual to assist in linkage to services as identified by the couple

Abbreviations: ART, antiretroviral therapy; CTR, counseling, testing, and referral; PACT, Protect and Connect; PEP, postexposure prophylaxis; PrEP, pre to exposure prophylaxis; STI, sexually transmitted infection.

The HIV CTR control condition included one 45-minute session consisting of individual rapid oral HIV or STI testing with pretest and posttest counseling and referrals to HIV or STI treatment and other social services. The HIV CTR control condition was selected to serve as a feasible, cost-effective comparison arm if no superior effects for the PACT intervention arm were found.

### Quality Assurance and Participant Flow

Intervention sessions in both arms were delivered by social workers or master’s-level social work students, all of whom completed a 4-day training on the intervention. We digitally recorded all sessions for both arms. The recording sessions were used for quality control and supervision. After listening to each session recording, the supervisors used the information gained to provide feedback to the facilitators to improve the fidelity and delivery of the sessions.

We screened 1167 individuals; 230 couples enrolled and were randomized into either the PACT intervention (115 couples) or HIV CTR control condition (115 couples). After we randomized the couples into the 2 arms, we delivered the first session. The 5 couple-based sessions for the PACT intervention condition were conducted weekly. Most couples finished the PACT intervention within 5 weeks after randomization; however, we allowed up to 2 months for couples to complete the intervention if they missed any sessions.

All control arm participants completed the single HIV CTR session. All PACT arm participants completed the first session, and 137 (59.6%) completed all 5 sessions; the mean (SD) number of sessions attended was 3.6 (1.8) sessions. Overall, follow-up retention rates, reflecting the participants who completed any of the follow-up assessments, were not statistically significantly different between arms (PACT arm = 208 [90.4%] vs HIV CTR control arm = 209 [90.9%]; *P* = .87). The retention rates at each follow-up assessment were as follows: PACT arm: 77.0% (177 participants) at 3 months, 84.3% (194 participants) at 6 months, and 87.8% (202 participants) at 12 months; HIV CTR control arm: 81.7% (188 participants) at 3 months, 83.5% (192 participants) at 6 months, and 83.5% (192 participants) at 12 months (Figure). The follow-up percentage increased over time, which may have been the result of the improved experience of the participants’ retention staff.

### Data Collection and Measurements

Biological assays for HIV infection and STIs were performed at baseline and at the 12-month follow-up. We also collected behavioral data at baseline and 3-, 6-, and 12-month follow-ups through audio computer-assisted self-interviews (ACASI) software. Participants received a maximum of $265 for all study activities, including session attendance, biological testing, and baseline and follow-up interviews. Sociodemographic variables were also collected. They included sex, age, race/ethnicity, marital status, years of education, employment status, monthly income, homelessness during the past 90 days, food insecurity in the past 90 days, and history of incarceration.

#### Biological Outcomes

We collected specimens using rapid oral HIV test (OraQuick ADVANCE Rapid HIV-1/2 Antibody Test; OraSure Technologies Inc). Women self-collected a vaginal swab specimen, and men provided a urine specimen to test for STIs. Specimens both men and women were assayed and tested for *Chlamydia trachomatis* and *Neisseria gonorrhoeae* using a DNA assay (BD ProbeTec ET Amplified DNA Assay; Becton, Dickinson and Co) and the women for *Trichomonas vaginalis* using a real-time polymerase chain reaction assay. We escorted participants with reactive HIV or positive STI test results to a physician or a department of health STIs clinic for confirmatory testing and/or treatment within 7 days. If 1 member of a couple had an STI-positive result at baseline, the partner with a negative result was also treated; thus, both partners were treated before the 12-month follow-up STI testing.

#### Primary and Secondary Behavioral Outcomes 

Self-reported data on sexual behaviors in the past 90 days were used to assess primary behavioral outcomes at all time points, including total number of times of vaginal and/or anal intercourse and the number of times of condomless vaginal and/or anal intercourse with the study partner and all other partners. We used Cronbach α, a measure for scale reliability, which indicated to what degree a set of items was associated with each other within a scale. Cronbach α is a function of the number of items, the mean intercorrelation between the pair items, and the mean variance of each item.

For the secondary behavioral outcomes, we assessed whether participants were under the influence of drugs or alcohol during the last (or most recent) time they had sex with their study partner, the number of sexual partners they had in the past 90 days, and whether they were tested for HIV in the past 90 days.

We also assessed whether participants had used substances ever and in the past 90 days (eg, binge drinking, using various illicit drugs, and injecting drugs), the number of days they did not use any drug in the past 30 days, and whether they attended an alcohol or drug treatment program in the past 90 days.

Condom use intentions were the sum of a 4-item Likert scale that assessed whether participants intended to use a male or female condom (0 indicated strongly disagree and 4 indicated strongly agree for each item response) and how often they planned to use a male or female condom (0 indicated never and 4 indicated every time for each item response) when they had sex with different partners in the next 3 months. Its scoring ranged from 0 (no intention) to 16 (high intention) with reliability of Cronbach α = .67 among the baseline participants. Condom use self-efficacy was a 5-item Likert scale that assessed participants’ confidence in using condoms with their study partner in challenging situations (0 indicated not at all confident and 4 indicated very confident for each item response). Its score was the sum of 5 items and ranged from 0 (not confident) to 20 (highly confident) with the scale reliability of Cronbach α = .94 at the baseline assessment. Couple safer-sex communication was assessed by the number of times participants reported discussing how they could prevent getting or transmitting HIV with their study partner in the past 90 days.

### Statistical Analysis

Statistical analyses were conducted from November 1, 2017, through June 1, 2018. For the cumulative STI incidence, we used a χ^2^ test for the difference in the incidence rates between the 2 study arms among participants who had STI-negative results at baseline or among those who had STI-positive results at baseline and both partners were confirmed to have received treatment.

For the behavioral outcomes, we followed an intent-to-treat approach and multiple imputation procedures to impute values for missing data for self-reported variables, including covariates and behavioral outcome variables, using the information that we observed or measured for a participant at previous assessments to predict values for variables that were missing.^21^ A total of 30 imputed data sets were generated.

Then, we used multilevel mixed-effects models and included a random effect for the dyad to account for dependencies within the couple, and another random effect for repeated measures. The models used the interaction between treatment condition and follow-up time, measures of the outcome variable reported at baseline, and other covariate adjustments (ie, sex, age, black or African American race/ethnicity, high school diploma or higher educational level, single status, homelessness, not having enough money for food, ever in prison, and illicit drug use). Hypothesis testing for intervention effects was based on incidence rate ratios (IRRs) from multilevel mixed-effects Poisson regression for the number of condomless sexual activities and other count variables and odds ratios (ORs) from multilevel mixed-effects logistic regression for whether participants were under the influence of drugs or alcohol when they last had sex with their study partners; the differences indicated by regression coefficients (β) from mixed-effects linear regression were used for the continuous secondary outcomes. Statistical significance was assessed using the associated 95% CI and 2-tailed *P* = .05 for each estimate. Multiple imputation procedures^21^ and statistical analyses were performed using Stata, version 14 (StataCorp LLC).

## Results

A total of 460 individuals (230 couples) were randomized into either the PACT intervention condition (230 individuals; 115 couples) or the HIV CTR control condition (230 individuals; 115 couples). Table 1 describes the sociodemographic characteristics and biological assays of STI status at baseline for the total sample and by intervention assignment. The mean (SD) age of participants was 35.0 (12.8) years; most participants self-identified as black or African American race/ethnicity (341 [74.1%]), had completed high school (291 [64.0%]), were single (271 [59.2%]), and were unemployed in the past 90 days (334 [72.9%]). Most participants reported having been arrested (362 [79.6%]), in jail (283 [62.2%]), or in prison (114 [24.9%]) in their lifetime. Almost half (211 [46.4%]) reported binge drinking, 407 (89.5%) had used illicit drugs, and 59 (13.0%) had injected drugs. Control participants were, in general, 5 years older than treatment participants, but no other substantial differences were found between conditions.

**Table 1.** Descriptive Statistics of the Sample Reported at the Baseline Assessment

Variable	No. (%)		
	Total Sample (N = 460)	HIV CTR Control Condition (n = 230)	PACT Intervention Condition (n = 230)
Age, mean (SD), y	35.0 (12.8)	37.7 (13.0)^a^	32.4 (12.1)^a^
Race/ethnicity			
Black or African American	341 (74.1)	178 (77.4)	163 (70.9)
Hispanic or Latino	87 (18.9)	39 (17.0)	48 (20.9)
High school diploma or higher educational level^b^	291 (64.0)	144 (63.2)	147 (64.8)
Single, never married^c^	271 (59.2)	131 (57.0)	140 (61.4)
Married or common law marriage^c^	157 (34.3)	81 (35.2)	76 (33.3)
Unemployed in the past 90 d^c^	334 (72.9)	164 (71.3)	170 (74.6)
Homeless in the past 90 d^c^	45 (9.8)	18 (7.8)	27 (11.8)
Not enough money for food in the past 90 d^c^	190 (41.5)	95 (41.3)	95 (41.7)
Ever arrested^b^	362 (79.6)	184 (80.7)	178 (78.4)
Ever in jail^b^	283 (62.2)	144 (63.2)	139 (61.2)
Ever in prison^c^	114 (24.9)	59 (25.7)	55 (24.1)
Ever binge drinking^b^	211 (46.4)	104 (45.6)	107 (47.1)
Binge drinking in the past 90 d^d^	131 (28.7)	64 (27.8)	67 (29.5)
Ever used illicit drugs^b^	407 (89.5)	205 (89.9)	202 (89.0)
Used illicit drugs in the past 90 d^d^	263 (57.6)	131 (57.0)	132 (58.2)
Ever injected drugs^b^	59 (13.0)	26 (11.4)	33 (14.5)
Injected drugs in the past 90 d^d^	28 (6.1)	13 (5.7)	15 (6.6)
HIV positive	32 (7.0)	17 (7.4)	15 (6.5)
Any STI	78 (17.0)	37 (16.1)	41 (17.8)
*Chlamydia trachomatis*	21 (4.6)	7 (3.0)	14 (6.1)
*Neisseria gonorrhea*	8 (1.7)	4 (1.7)	4 (1.7)
*Trichomoniasis vaginalis*	51 (11.1)	28 (12.2)	23 (10.0)

Abbreviations: CTR, counseling, testing, and referral; PACT, Protect and Connect; STI, sexually transmitted infection.

^a^ *P* < .01; tests of difference between condition assignments by unpaired, 2-tailed *t* test or χ^2^ test.

^b^ Five missing values.

^c^ Two missing values.

^d^ Three missing values.

At baseline, 32 participants (7.0%) had reactive HIV rapid tests (5 men and 10 women in the PACT arm, and 7 men and 10 women in the CTR control arm), and 78 (17.0%) had a positive test result for any STI (16 men and 25 women in the PACT arm, and 10 men and 27 women in the CTR control arm). No new HIV infections were detected at the 12-month follow-up. Among the participants who had a negative result for any STI at baseline, or who had positive STI results but both partners completed treatment, 18 new STIs were identified at the 12-month assessment, including 10 (6.4%) from the PACT arm (3 men and 7 women) and 8 (5.8%) from the CTR control arm (8 women). However, these differences in new STI cases between the 2 arms were not statistically significant.

Table 2 presents descriptive statistics regarding primary and secondary outcomes at the baseline and each follow-up assessment by intervention assignment. The results of multilevel mixed-effects models in Table 3 show that during the entire 12-month period, PACT participants had 33% fewer acts of condomless vaginal and/or anal intercourse with their study partners in the past 90 days (IRR, 0.67; 95% CI, 0.45-0.99; *P* = .04), 70% fewer acts with other partners (IRR, 0.30; 95% CI, 0.12-0.74; *P* = .009), and 40% fewer acts with all sexual partners (IRR, 0.60; 95% CI, 0.42-0.85; *P* = .005) compared with control participants. Significantly fewer acts of condomless intercourse among PACT participants were also found at each follow-up assessment.

**Table 2.** Descriptive Statistics of Primary and Secondary Outcome Variables at the Baseline and the Follow-up Assessments

Outcome	Condition	Baseline Assessment (n = 456)^a^	Follow-up Assessment		
			3 mo (n = 365)	6 mo (n = 385)	12 mo (n = 392)
**Primary Outcomes**					
No. of times of condomless vaginal and/or anal intercourse, mean (SD)					
With study partner in the past 90 d	HIV CTR	28.3 (34.8)	18.5 (29.1)	18.2 (30.0)	9.4 (21.6)
	PACT	27.6 (33.5)	15.6 (25.6)	16.2 (30.2)	7.3 (18.4)
With non–study partner(s) in the past 90 d	HIV CTR	1.8 (8.4)	1.5 (7.5)^b^	1.1 (6.9)	2.1 (10.0)
	PACT	1.3 (7.1)	0.3 (1.9)^b^	0.9 (7.1)	1.1 (7.2)
With all partners in the past 90 d	HIV CTR	30.1 (35.4)	19.9 (30.2)	19.2 (31.5)	11.4 (23.3)
	PACT	28.9 (35.5)	15.9 (25.5)	17.1 (32.3)	8.4 (19.5)
**Secondary Outcomes**					
Last (most recent) time of vaginal and/or anal intercourse with study partner was under the influence of drugs or alcohol, No. (%)	HIV CTR	82 (36.0)	47 (25.0)^b^	43 (22.5)	37 (19.5)
	PACT	92 (40.4)	29 (16.4)^b^	35 (18.0)	32 (15.8)
No. of sexual partners in the past 90 d, mean (SD)	HIV CTR	2.0 (3.7)	1.9 (3.9)^c^	2.0 (6.3)	1.3 (1.6)
	PACT	1.7 (1.9)	1.0 (0.6)^c^	1.1 (1.2)	1.0 (1.4)
Condom use intentions, Likert scale score, mean (SD)^d^	HIV CTR	11.6 (3.6)	11.7 (4.0)^c^	12.1 (3.7)	11.8 (4.2)^b^
	PACT	12.1 (3.4)	12.8 (3.4)^c^	12.8 (3.6)	12.7 (3.7)^b^
Condom use self-efficacy, Likert scale score, mean (SD)^e^	HIV CTR	9.9 (7.4)	11.8 (7.7)^b^	12.4 (7.6)^b^	11.9 (7.6)^c^
	PACT	10.8 (7.2)	13.5 (7.0)^b^	14.1 (6.9)^b^	14.0 (7.1)^c^
No. of times discussed with study partner how to prevent HIV infection in the past 90 d, mean (SD)	HIV CTR	9.7 (24.7)	7.0 (19.1)^b^	7.1 (20.4)	6.3 (18.6)
	PACT	7.9 (21.7)	12.1 (27.5)^b^	10.3 (25.2)	7.0 (20.5)
Had HIV testing in the past 90 d, No. (%)	HIV CTR	127 (55.2)	105 (55.9)	90 (46.9)	65 (33.9)
	PACT	142 (62.3)	98 (55.4)	93 (47.9)	76 (37.6)
Injected drugs in the past 90 d, No. (%)	HIV CTR	13 (5.7)	13 (7.2)	12 (6.3)	15 (7.8)
	PACT	15 (6.6)	15 (8.6)	14 (7.3)	16 (7.9)
Entered drug treatment in the past 90 d, No. (%)	HIV CTR	26 (11.4)	35 (18.7)	23 (12.0)	24 (12.5)
	PACT	27 (12.1)	28 (15.8)	24 (12.4)	21 (10.4)

Abbreviations: CTR, counseling, testing, and referral; PACT, Protect and Connect intervention.

^a^ Four missing responses at the baseline assessment.

^b^ *P* < .05.

^c^ *P* < .01.

^d^ Likert scale assessed whether participants intended to use a male or female condom (0 indicated strongly disagree and 4 indicated strongly agree for each item response) and how often they planned to use a male or female condom (0 indicated never and 4 indicated every time for each item response) when they had sex with different partners in the next 3 months. Its scoring ranged from 0 (no intention) to 16 (high intention).

^e^ Condom use self-efficacy was a 5-item Likert scale that assessed participants’ confidence in using condoms with their study partner in challenging situations (0 indicated not at all confident and 4 indicated very confident for each item response). Its score was the sum of 5 items and ranged from 0 (not confident) to 20 (highly confident).

**Table 3.** Multilevel Models for Intervention Effect Estimates at Each Follow-up Assessment and During the Entire Follow-up Period After Multiple Imputation (30 Imputed Data Sets)^a^

Outcome	Entire Follow-up Period	Follow-up Assessment		
		3 mo	6 mo	12 mo
**Primary Outcomes**				
No. of times of condomless vaginal and/or anal intercourse, IRR (95% CI)				
With study partner in the past 90 d	0.67 (0.45 to 0.99)^b^	0.71 (0.48 to 1.04)	0.67 (0.46 to 0.99)^b^	0.61 (0.41 to 0.91)^b^
*P* Value	.04	.08	.04	.02
With non–study partner(s) in the past 90 d	0.30 (0.12 to 0.74)^c^	0.33 (0.13 to 0.86)^b^	0.31 (0.12 to 0.77)^b^	0.26 (0.10 to 0.66)^c^
*P* Value	.009	.02	.01	.004
With all partners in the past 90 d	0.60 (0.42 to 0.85)^c^	0.64^b^ (0.45 to 0.92)	0.60 (0.42 to 0.86)^c^	0.53 (0.37 to 0.76)^c^
*P* Value	.005	.02	.005	.001
**Secondary Outcomes**				
Last (most recent) time of vaginal and/or anal intercourse with study partner was under the influence of drugs or alcohol, OR (95% CI)	0.55 (0.31 to 0.96)^b^	0.60 (0.30 to 1.20)	0.56 (0.31 to 0.99)^b^	0.47 (0.23 to 0.99)^b^
*P* value	.04	.15	.046	.046
No. of sexual partners in the past 90 d, IRR (95% CI)	0.74 (0.61 to 0.88)^c^	0.69 (0.55 to 0.87)^c^	0.73 (0.61 to 0.87)^c^	0.81 (0.64 to 1.03)
*P* Value	.001	.002	.001	.09
Condom use intentions, Likert scale, β (95% CI)^d^	0.71 (0.15 to 1.26)^b^	0.70 (0.02 to 1.38)^b^	0.71 (0.14 to 1.27)^b^	0.71 (−0.02 to 1.44)
*P* Value	.01	.04	.01	.06
Condom use self-efficacy, Likert scale, β (95% CI)^e^	1.52 (0.42 to 2.62)^c^	1.35 (0.03 to 2.67)^b^	1.48 (0.36 to 2.60)^c^	1.74 (0.34 to 3.13)^b^
*P* Value	.007	.046	.01	.02
No. of times discussed with study partner how to prevent HIV infection in the past 90 d, IRR (95% CI)	1.89 (1.06 to 3.36)^b^	2.18 (1.22 to 3.89)^c^	1.93 (1.09 to 3.43)^b^	1.52 (0.84 to 2.75)
*P* Value	.03	.009	.03	.16

Abbreviations: IRR, incidence rate ratio; OR, odds ratio.

^a^ Multilevel models included random effects for couple, repeated measures, and covariate adjustments for baseline measures of the outcomes (sex, age, black or African American race/ethnicity, high school, single, homeless, not enough money for food, ever in prison, and illicit drug use).

^b^ *P* < .05.

^c^ *P* < .01.

^d^ Likert scale assessed whether participants intended to use a male or female condom (0 indicated strongly disagree and 4 indicated strongly agree for each item response) and how often they planned to use a male or female condom (0 indicated never and 4 indicated every time for each item response) when they had sex with different partners in the next 3 months. Its scoring ranged from 0 (no intention) to 16 (high intention).

^e^ Condom use self-efficacy was a 5-item Likert scale that assessed participants’ confidence in using condoms with their study partner in challenging situations (0 indicated not at all confident and 4 indicated very confident for each item response). Its score was the sum of 5 items and ranged from 0 (not confident) to 20 (highly confident).

In addition, PACT participants were less likely to report being under the influence of drugs or alcohol the last time they had vaginal and/or anal intercourse with their study partners (OR, 0.55; 95% CI, 0.31-0.96; *P* = .04) and had 26% fewer sexual partners in the past 90 days (IRR, 0.74; 95% CI, 0.61-0.88; *P* = .001) compared with control participants over the entire follow-up period. Significant increases were found in condom use intentions, condom use self-efficacy, and safer sex communication when comparing PACT participants with their control counterparts (Table 3). No statistically significant difference between the 2 arms was found with regard to injection drug use, additional HIV testing, and entry into drug or alcohol treatment.

We performed multilevel analyses using the complete cases (ie, only observed data) with and without covariance adjustments. Both results yielded the similar variable estimates and statistically significant patterns as found with the multiple imputation analyses. In addition, we used the complete cases and conducted the multilevel analyses with bootstrapping for 200 replications. These findings were also consistent with the findings from the multiple imputation analyses.

## Discussion

This RCT demonstrated that PACT’s couple-based 5-session intervention can lower self-reported sexual risk behaviors among men in CSPs and their female sexual partners, including substantial reductions in condomless intercourse, the number of sexual partners, and by not being under the influence of drugs or alcohol the last time they had sex. Increasing condom use over time is important because condom use remains the key HIV or STIs prevention tool to diminish sexual transmission. A Cochrane Collaboration systematic review showed that consistent, proper condom use is associated with an 80% reduction in HIV incidence among HIV-discordant heterosexual couples.^22,23^ Participants randomized to receive the PACT sessions showed significant improvement in condom use intention, condom use self-efficacy, and safer sex communication, compared with CTR control participants. These 3 outcomes were found to be important factors in sexual risk reduction.^13,18^ The overall consistency, strength, and magnitude of the outcomes strengthen confidence in the effectiveness of the PACT intervention at 12 months (the time frame used in this study), and these findings support previous studies. A systematic review of HIV interventions showed that all of the previous couple-based studies used a 12-month follow-up.^7^ None of these studies examined sustainability beyond this time frame. One major conclusion of the systematic review was that those who learned new behaviors and sustained them for 12 months were likely to continue those behavioral changes for a longer period^7^; however, this issue has not been tested.

In contrast to the significant effects observed for the behavioral outcomes, the PACT intervention did not influence the cumulative incidence of STIs. During the follow-up period, we observed no new HIV cases, assessed using the rapid oral HIV test. We closely followed the trial’s STI treatment referral protocol for participants in both arms to ensure that all participants with STI-positive results and their study partners obtained treatment and adhered to the STI treatment regimen. After the STI cases found at baseline were treated, 18 additional STI cases appeared, indicating a continuance of risky behaviors. However, most participants who had STI-positive results at baseline or follow-up were treated and became disease free. The lack of support for biological STI outcomes, despite strong findings in support of behavioral outcomes, is consistent with all but 1 HIV or STI prevention interventions that have met the criteria for inclusion in the Centers for Disease Control and Prevention’s portfolio of evidence-based interventions^24^; this lack of support remains a challenge for future studies.

These findings highlight the importance of shifting the HIV prevention focus from individual-oriented approaches to couple-based approaches. Research calls for additional couple-based HIV interventions tailored to people mandated to be in a CSP and for implementation strategies to promote the delivery of couple-based approaches in CSPs, in which most participants have been incarcerated more than once and recidivism among this population is high.^25^ Incarceration often leads to the dissolution of existing sexual partnership,^26^ which increases the risks of engaging in sexual and drug-use behaviors, including overdose.^26,27,28,29^ The PACT intervention sessions are designed to address each of these issues.

### Strengths and Limitations

This study has a number of strengths. The strengths are high rates of participation, attendance, and retention over the 12-month follow-up. In addition, the intervention was delivered in real-world CSP settings.

This study also has several limitations. Behavioral outcomes used validated measures, but the responses were self-reported and may have biased study findings. Generalizability of study findings is limited to men in CSPs in New York City. Moreover, some of the men who were interested in participating in the study reported that their female sexual partners did not want to participate because of a lack of commitment to staying together or because they were too busy to attend the sessions. Without his sexual partner, the interested man was disqualified from participating in the study. This situation may have caused us to include couples who were motivated to stay together and eager to learn ways to enhance their relationship and reduce drug and sexual risks, compared with those who were screened out. This bias has a potential limitation in the conclusion, in which the findings may not be generalizable to all men in CSPs, given that 29% of the men screened were not included because they were unable to bring in their female partners for screening.

## Conclusions

This RCT demonstrated that a 5-session intervention delivered to men in CSPs and their female sexual partners can significantly reduce self-reported sexual risk behaviors, including condomless intercourse, the number of sexual partners, and being under the influence of drugs or alcohol the last time they had sex. The male participants were willing to invite their main female partners to enroll in the study, and study participation and retention were high. These findings underscore that the PACT intervention can be scaled up to curb the burgeoning HIV epidemic in CSPs and similar criminal justice settings.

## Notes

Supplement 1. **Trial Protocol**

Supplement 2. **eAppendix**. Eligibility and Exclusion Criteria

Supplement 3. **Data Sharing Statement**
