# Supplementary material for: Effectiveness of a Couple-Based HIV and Sexually Transmitted Infection Prevention Intervention for Men in Community Supervision Programs and Their Female Sexual Partners: A Randomized Clinical Trial
Source: JAMA Netw Open. 2019 Mar 29;2(3):e191139. doi: 10.1001/jamanetworkopen.2019.1139 (PMC6450427; doi:10.1001/jamanetworkopen.2019.1139)
Supplement: Supplement 1. — Trial Protocol [file jamanetwopen-2-e191139-s001.pdf]

PACT STUDY PROTOCOLS

Title of Project: Protect and Connect: Couple HIV Prevention for Drug Involved Male Offenders (PACT)

Date Protocol submitted to clinicaltrails.gov: June 11, 2013

Team of investigators:

Principle Investigator - Nabila El-Bassel, PhD
Co- Investigator - Louisa Gilbert, PhD
Co Investigator - Elwin Wu, PhD
Co- Investigator - Timothy Hunt, PhD

ClinicalTrials.gov Identifier: NCT01690494

Reference:
El-Bassel, N., Gilbert, L, Wu, E. and Hunt, T. (June 11, 2013). Couple HIV Prevention for Drug Involved Male Offenders (PACT): Clinical Trial Protocol. ClinicalTrials.gov identifier: NCT01690494. https://clinicaltrials.gov/ct2/show/study/NCT01690494?cond=couples+hiv&rank=3

Intervention:
The HIV CTR control condition includes case manager services to provide referrals to drug treatment, educational employment, and social services. Typically these services at the community correctional program sites are provided in 4 or fewer contacts. After an initial intake and case management referrals couples who are assigned to the HIV CTR control condition at randomization will receive a handbook of services designed for drug-involved male offenders to ensure that they have received basic service information. In addition, all couples assigned to HIV will receive Respect Brief HIV/STI pre-test or post-test counseling and referrals at their baseline visit. Providing evidence-based HIV/STI testing, counseling and referrals to all control participants at baseline will not only improve scientific rigor of the study but also ensure that the HIV CTR control condition meets ethical standards as a comparison group. At all assessments, data will be collected on number and type of visits and any other service or information received from participants and court records to monitor intensity and implementation of common elements of HIV CTR.

The PACT condition is delivered in community correctional program sites. Male offender participants who are assigned to PACT will also continue to receive regular services. The intervention is conducted by trained study staff. PACT addresses both drug and sexual risks with the following core components: (1) couple-based HIV Counseling Testing and Referral (CTR), (2) disclosure of drug and sexual risks; (3) couple communication, negotiation, and problem-

solving skills to reduce drug and sexual risks; (4) technical condom use skills; (5) strategies for reducing unsafe injections; (6) biomedical HIV prevention strategies (HIV treatment as prevention, PEP, PrEP; (7) linkage to HIV, STI and substance use treatment; (8) reproductive health issues (9) risks and experiences of sexual coercion, (10) risks for opioid overdose; (11) informal social support; and (12) couple goal setting to reduce long-term HIV risks.

PACT is designed to be educational, engaging, and tailored to the realities of drug-involved couples in CCP settings, through role play scenarios that highlight skills needed to face common challenges (e.g., coping with mandates and stigma). Sessions last one hour and will be conducted twice a week with the same case manager. For each session, couples will use a resource manual to help them identify and prioritize their HIV/STI, drug abuse and other service needs and to link them to appropriate services using a web-based program with an action plan for service engagement for each session.

Recruitment, Screening and Eligibility:

Trained RAs will approach male clients at CCP sites after they are sentenced and give them flyers about the study. If a man is receptive, the RA will describe the study and answer any questions. If the man indicates that he is interested in the study, the RA will obtain consent to be screened, conduct a screening interview to determine eligibility and willingness to participate. The screening interview will contain eligibility related items, socio-demographics, and other items that will camouflage the eligibility criteria. Once the potential participant's eligibility has been established, he will be asked to invite his main female sexual partner to participate. If a potential participant and his partner inform the RA that they agree to participate, both are consented and screened. If the couple meets eligibility criteria, the RA will schedule the couple for a baseline assessment. We will provide monetary compensation to participants for completing the screening and all assessments

<u>Eligibility Inclusion Criteria</u>: Couples are eligible to participate if: (i1) both partners were aged 18 or older; (i2) both partners identified each other as their primary sexual partner of the opposite sex; (i3) the relationship had lasted at least 3 months; (i4) at least one partner reported having had condomless vaginal and/or anal intercourse with the other in the past 90 days; (i5) at least one partner reported exposure to an outside HIV risk in the past year (i.e., engaged in unprotected sex with another partner, shared syringes, tested positive for an HIV/STI) or at least one partner suspected that their partner had sex with another partner, injected drugs, was HIV positive or had tested positive for an STI ; (i6) the couple planned to stay together for at least another year; (i7) the male partner reported either (a) use of illicit drugs or binge drinking (i.e. drinking 5 or more alcoholic beverages) in the past 90 days or (b) attended substance abuse treatment in the past 90 days; and (i8) the male partner was mandated to community supervision, alternative to incarceration or probation verified by court records.

<u>Eligibility Exclusion Criteria</u>: Couples are excluded if: (e1) either partner shows evidence of significant psychiatric, physical or neurological impairment that would limit effective participation as confirmed during informed consent; (e2) either partner reported an order of protection within the past year or identified any safety concerns about participating in sessions with their partner; or; (e3) either partner did not have sufficient fluency in English.

Rationale for Eligibility Criteria: This study includes HIV positive, HIV negative, and HIV serodiscordant couples as this intervention has been designed for and tested with mixed status couples with a focus on dyadic strategies to keep HIV negative partners from seroconverting and to promote HIV treatment and ARV medication adherence among HIV positive partners. The study is limited to heterosexual relationships because our couple-based research with men who have sex with men indicate that gender roles and risk environments are likely to substantially differ.

Overview of subject participation:
In the initial contact, a research assistant (RA) will administer consent and screen potential participants and their partners to determine their potential eligibility. Those couples deemed eligible are scheduled for the baseline visit, within 30 days of the initial screening. At the baseline visit, after the couple's eligibility is reconfirmed and the RAs administer the informed consent. Participants are then escorted to a private room and given instructions on the Audio Computer Assisted Self-Interview (ACASI) by the RA. After the baseline ACASI interview is completed, the RA conducts individual pre-test counseling for HIV and STIs and post-test counseling for HIV to each participant. Specimens are then collected, stored and shipped. The Clinical Research Coordinator will notify each participant privately of his or her STI test results within 7-10 days of the baseline visit, provide post-test counseling, and refer to STI treatment (if applicable). Within 1-10 days of their baseline visit, couples will be randomized to PACT or the HIV CTR control and the first PACT session will occur. Couples assigned to the HIV CTR control will receive a service handbook and service referrals. Intervention sessions 2-4 for PACT are implemented twice weekly at the CCP sites. The 3-month follow up will take place within 80-110 days of the last intervention session, which could range from 10-14 days post randomization for PACT condition. For the HIV CTR control condition, this date will be set at 12 days post randomization (mid midpoint of this range). The 6-month assessment will occur within 180 to 210 days of the last session. The 12-month assessment will take place within 360-390 days of the last session. At each follow up assessment, participants complete the ACASI interview. At the 6 and 12-month assessments biospecimens will be collected to test for STIs. At the 12-month follow-up, HIV negative participants will be retested for HIV.

Retention:
We employ several steps to ensure retention from first point of screening to final 12-month follow-up that we have used successfully in previous research with drug-involved offenders under community supervision, including (1) providing compensation for assessments and reimbursing participants for transportation at each intervention session; (2) collecting detailed locator information, contacting participants before each session, and maintaining biweekly contact with participants during the follow-up period; (3) tracking participants who are incarcerated or in drug-treatment..

Assessment:
Primary outcomes include indicators of sexual and drug risk based on self-reported behaviors and objective biological assay for STIs. We utilize a modified RBA to capture different types of sexual behaviors with study partners as well as with all other partners in the past 90 days. Secondary outcomes of drug use, access to drug treatment and other services, and recidivism will

be based on self-report and public court records. Intervention mediator and moderator measurements are based on self-report, organizational level data collected from CCP management staff, frontline providers and/or administrators. GIS community level data will be collected using zip code or census tract data from publically available databases. Service mapping of all drug treatment, social services and HIV prevention, testing and treatment services in NYC will be regularly updated in GIS data set. These instruments have been used in the baseline ACASI interview will take 75 minutes on average for participants to complete and the follow up assessments will take 40 minutes.

<u>Assay for HIV, chlamydia, trichomonas and gonorrhea</u>
For biological assays for HIV, we collect an oral specimen using OraQuick ADVANCE Rapid HIV. For chlamydia, trichomonas and gonorrhea, women self-collect a vaginal swab specimen and men provide a urine specimen. Specimens are assayed and tested for Chlamydia trachomatis and Neisseria gonorrheae using the Becton Dickinson Probe ET Amplified DNA Assay (Becton, Dickinson and Co, Sparks, Maryland) and for Trichomonas vaginalis using a real-time polymerase chain reaction assay.

<u>Costs</u> are estimated based on material purchase/consumption and time recorded regularly as part of project records. Additional data necessary for cost calculations, such as salary and capital costs (e.g., rent), will be obtained during administrative review, performed annually to account for inflation, from figures available and obtained by the Center for Court Innovation and the NYC Dept. of Probation.

Data Analyses:
The analytic approach is built upon the investigative team's prior HIV prevention randomized clinical trials. An intention-to-treat approach will be used. To enhance the rigor of an intention-to-treat analytic approach, we will employ multiple imputation to handle missing data, attrition analyses to investigate non-ignorable nonresponse, and sensitivity analyses to ascertain robustness of findings against violation of model assumptions (e.g., distribution requirements, heteroscedasticity, independence). Multiple imputation will be implemented using the imputation by chained equations algorithm (ice) in the statistical software Stata. This procedure allows specifying linear regression to impute continuous variables, logistic regression to impute dichotomous variables, multinomial logistic regression to impute categorical variables, and negative binomial regression to impute count data. Specifications of the imputation model will be based on the information that is observed or measured for a participant at prior assessments to predict values for variables that are missing, including covariates from the baseline assessment and behavioral outcome variables at the baseline and each follow-up assessment. The analysis plan is also informed by earlier RCTs of couple-based interventions. Generalized Linear Mixed Models (GLMM) will be used to account for the non-independence in measures arising from: (1) "autocorrelation" due to repeated measures with the same person; and (2) "intraclass correlation [ICC]" arising from partners who are reporting on conjoint behaviors. These models build upon $g\left(E\left[\left(Y_{ij}\middle|\mathbf{x}_{ij}\right)\right]\right) = \mathbf{x'}_{ij}\boldsymbol{\beta}$ where Y_{ij} is the outcome variable of interest for the ith participant at the jth timepoint. The multilevel nature of the data are accounted for by modeling each predictor variable's coefficient in the vector $\boldsymbol{\beta}$ as dependent on couple membership: $\beta_i = \mathbf{w}_g\boldsymbol{\gamma}$ where the ith participant is part of the gth couple which determines the couple characteristics (i.e., elements of the vector $\mathbf{w}$) used to estimate model fit parameters in vector $\boldsymbol{\gamma}$. The components of $\boldsymbol{\beta}$ will be

treatment assignment, baseline attributes for the outcome variable, and those variables selected during preliminary analyses as noted earlier. The g(a) function represents a link function that can properly model the outcome variable as a function of measurement unit or distribution. Hypothesis testing follows the form "Assignment to PACT will be associated with significantly lower/higher on outcome variable Y at the 12-month follow-up compared to assignment to HIV CTR" where 'lower' or 'higher' is chosen in the direction of reduced risk. Outcome-specific modeling information for hypothesis testing is presented in Table 1.

Table 1: Data Analytic Approach for Hypotheses Testing

	Y	Candidate* link function $\mathbf{g(a)}$	Covariates used to estimate $\boldsymbol{\beta}$ & $\boldsymbol{\gamma}$
H_1	• Incidence of Chlamydia, trichomonas and gonorrhea	• Logistic: $\log(a/(1-a))$<br>• Poisson: $\log(a)$	• Baseline attributes for sociodemographics<br>• Attribute(s) of Y measured at baseline
H_2	• # of acts of unprotected intercourse	• Poisson: $\log(a)$<br>• Negative Binomial: $\log(a/(k+a))$	
H_3	• # of sexual partners	• Poisson: $\log(a)$<br>• Negative Binomial: $\log(a/(k+a))$	
H_4	• # of days in which no illicit drugs were used	• Poisson: $\log(a)$<br>• Negative Binomial: $\log(a/(k+a))$	
H_5	• Drug Treatment Attendance/Engagement	• Logistic: $\log(a/(1-a))$	

Candidate link functions (beyond distributional requirements) will be determined by selecting the function with the best overall fit metrics followed by statistical efficiency of the coefficient for study arm assignment.

Power Analysis: Power analyses were conducted using G*Power (v3.1.0). Autocorrelation (ρ) and ICC estimates were based on Project PACT trial and Pilot of Revised PACT in CCP settings. From ICC estimates, variance inflation factors were calculated to generate effective sample sizes. Outcome estimates for intervention effects were obtained from Project PACT while those for HIV CTR used PACT wellness promotion comparison condition as a proxy for non-specific attention. These estimates allow for effect size f^2 to be calculated. For cumulative STI incidence, we assumed a 50% reduction to be clinically meaningful to detect via Poisson regression. Results for primary outcomes indicate that a minimum of 80% power is achieved for all primary outcomes with 96 couples per arm. Based on the team's successful retention in Project WORTH with drug-involved offenders and prior couple-based studies for the likelihood of 80% to 85% retention, this is corresponding to 113 to 120 couples per arm (or a total of 226 to 240 couples) before 15% to 20% attrition. Power analyses with 96 participants per arm indicate 80% power to detect differences for effect sizes $f^2 = .03$ to .04 (for $\rho = .1$ TO .5), indicating the ability to detect substantially smaller than "medium" effect sizes (Cohen, 1988).
