# Supplementary material for: Effectiveness of a Couple-Based HIV and Sexually Transmitted Infection Prevention Intervention for Men in Community Supervision Programs and Their Female Sexual Partners: A Randomized Clinical Trial
Source: JAMA Netw Open. 2019 Mar 29;2(3):e191139. doi: 10.1001/jamanetworkopen.2019.1139 (PMC6450427; doi:10.1001/jamanetworkopen.2019.1139)
Supplement: Supplement 2. — eAppendix. Eligibility and Exclusion Criteria [file jamanetwopen-2-e191139-s002.pdf]

Supplementary Online Content

El-Bassel N, Gilbert L, Goddard-Eckrich D, et al. Effectiveness of a couple-based HIV and sexually transmitted infection prevention intervention for men in community supervision programs and their female sexual partners: a randomized clinical trial. *JAMA Netw Open*. 2019;2(3):e191139. doi:10.1001/jamanetworkopen.2019.1139

eAppendix. Eligibility and Exclusion Criteria

This supplementary material has been provided by the authors to give readers additional information about their work.

© 2019 El-Bassel N et al. *JAMA Network Open.*

eAppendix. Eligibility and Exclusion Criteria.

Eligibility criteria for the study

1. Both partners were aged 18 or older
2. Both partners identified each other as their primary sexual partner of the opposite sex
3. The relationship had lasted at least 3 months
4. At least one partner reported having had condomless vaginal and/or anal intercourse with the other in the past 90 days
5. At least one partner reported exposure to an outside HIV risk in the past year (engaged in unprotected sex with another partner, shared syringes, tested positive for an HIV/STI) or at least one partner suspected that their partner had exposure to an outside HIV risk
6. The couple planned to stay together for at least another year
7. The male partner reported either (a) use of illicit drugs or binge drinking (i.e., drinking 5 or more alcoholic beverages on a single occasion) in the past 90 days or (b) attended substance abuse treatment in the past 90 days
8. The male partner was mandated to community supervision, alternative to incarceration or probation verified by court records

Couples were excluded if

1. Either partner showed evidence of significant psychiatric or cognitive impairment as assessed during informed consent
2. Either partner reported an order of protection within the past year or identified any safety concerns about participating in sessions with their partner
3. Either partner did not have sufficient fluency in English

© 2019 El-Bassel N et al. *JAMA Network Open*.
