# Supplementary material for: Effectiveness of a Couple-Based HIV and Sexually Transmitted Infection Prevention Intervention for Men in Community Supervision Programs and Their Female Sexual Partners: A Randomized Clinical Trial
Source: JAMA Netw Open. 2019 Mar 29;2(3):e191139. doi: 10.1001/jamanetworkopen.2019.1139 (PMC6450427; doi:10.1001/jamanetworkopen.2019.1139)
Supplement: Supplement 3. — Data Sharing Statement [file jamanetwopen-2-e191139-s003.pdf]

Data Sharing Statement

El-Bassel. Effectiveness of a Couple-Based HIV and Sexually Transmitted Infection Prevention Intervention for Men in Community Supervision Programs and Their Female Sexual Partners. *JAMA Netw Open*. Published March 29, 2019.
10.1001/jamanetworkopen.2019.1139

Data
Data available: Yes
Data types: Deidentified participant data
How to access data: Data will be made available in Academic Commons, Columbia University's Data Repository program.
When available: With publication

Supporting Documents
Document types: None

Additional Information
Who can access the data: To anyone requesting the data.
Types of analyses: For any purpose.
Mechanisms of data availability: Please refer to the terms of Columbia's Academic Commons for more information.
